# Alum: an old dog with new tricks

**DOI:** 10.1038/emi.2016.40

**Published:** 2016-03-23

**Authors:** Yumei Wen, Yan Shi

**Affiliations:** 1 Key Laboratory of Molecular Medical Virology, MOE/MOH, Shanghai Medical College, Fudan University, Shanghai 200032, China; 2 Department of Basic Medical Sciences, Institute of Immunology, Center for Life Sciences, Tsinghua University, Beijing 100084, China; 3 Department of Microbiology, Immunology and Infectious Diseases, University of Calgary, Calgary T2N 4N1, Alberta, Canada

**Keywords:** alum, cancer therapy, crystals, hepatitis B, inflammasome

## Abstract

Aluminum compounds (alum) are the most widely used adjuvants in veterinary and human vaccines. Alum was initially thought to be a simple depot for antigen retention; however, our understanding of the mechanism by which it works has progressed substantially in recent decades. Nonetheless, consensus regarding its roles in different aspects of immune regulation has not been reached, and it remains a long-standing research subject in the field of vaccinology. This review, in chronological order, discusses the various hypotheses proposed in mostly inadequate attempts to illuminate the mechanism by which alum works, from the depot theory to the involvement of the NLRP3 inflammasome and from cell death-associated danger factors to crystalline structure-mediated plasma membrane alteration. In addition, novel findings of unexpected beneficial effects of decreased HBV (Hepatitis B virus) viral load and HBeAg seroconversion in chronically infected patients, as well as significant tumor suppression in experimental mice following multiple alum-only injections are examined, revealing alum's potential clinical applications beyond its use as a simple tool in antigen preparation. With increasing threats of emerging microbes, originating from natural or man-made sources, that pose significant health concerns at the population scale, the potential use of alum as a ‘first-aid' vaccine is also discussed.

## INTRODUCTION

Aluminum compounds (alum) have been used as adjuvants for nearly 90 years in veterinary and human vaccines. Alum facilitates effective and long-lasting protective immunity in hosts, mainly by inducing antibody responses. Although human vaccine preparations have seen considerable progress in recent years, alum remains the most widely used adjuvant. The adjuvant effect of alum was first described by Glenny *et al.*,^1^ who serendipitously found that in processing an immunogen, diphtheria toxoid, by precipitation with potash (crude KAl(SO_4_)_2_·12H_2_O), the resulting mixture induced a significantly increased immune response against the toxoid. It was subsequently found that protein preparations precipitated with alum were highly heterogeneous, depending on which anions, such as bicarbonate, sulfate or phosphate, were present at the time of precipitation. To improve and optimize the protein–alum preparation, Maschmann *et al.*^2^ used aluminum gels that could be preformed using a well-defined method to adsorb protein antigens in aqueous solution, representing a major step forward for standardizing the use of alum as common adjuvants. Vaccine preparations based on this approach are called aluminum-adsorbed vaccines, in contrast to alum-precipitated vaccines used in earlier days. Starting in the late 1940s, highly uniform aluminum hydroxide became available and was used as an adjuvant in a large number of vaccines. Aluminum phosphate was introduced somewhat later. For instance, for the preparation of active diphtheria toxoid, equal molarities of aluminum chloride and trisodium phosphate were used to yield pure aluminum phosphate as the resulting adsorbent.^3^ Although hydroxide and phosphate salts remain the primary choice, new adjuvants are being developed for different immunization purposes.^4, 5, 6^ Despite its popularity in practice, the mechanisms underlying the adjuvant functionality of alum are still being discovered and represent an interesting topic in vaccinology.

## MECHANISMS OF ALUM AS AN ADJUVANT

### Challenges against the ‘depot theory'

The earliest idea regarding the immune regulatory activities of alum was based on an observation by Glenny himself, who reported that the precipitation of antigen onto insoluble particles of aluminum potassium sulfate before immunization produced better antibody responses than soluble antigen alone. He noted that the clearance of alum-precipitated toxoid from the injection site was delayed in comparison with plain toxoid in immunized guinea pigs. This observation led him to believe that the association with an aluminum salt served a ‘depot' effect, which could enhance the immune response via antigen retention, thus establishing a prolonged release phase from the low solubility salt. This dogmatic explanation was rarely challenged until the last two decades, when a number of scientists started to reveal the multiple immunological roles played by alum.

It was shown that removal of the antigen-aluminum salt nodules 14 days after immunization had no effect on subsequent antibody titers, indicating that alum preparations do not function simply by providing a long-lived antigen depot.^7^ Furthermore, experiments showed that immunization of rabbits with antigen plus alum induced the appearance of B-cell lymphoblasts in the draining lymph nodes within seven days and at the site of the granuloma by day 14. After three weeks, few B cell lymphoblasts remained in the draining lymph nodes, inviting questions on the necessity of long-term antigen presentation beyond two weeks.^8^ More recent studies further suggest that alum admixed antigens appeared in draining lymph nodes within several hours to a day, and their migration did not last beyond this period. Interestingly, removal of the injection site (i.e., ear) 2 h after antigen delivery had no effect on subsequent CD4^+^ T-cell activation or division.^9^ In addition, alum's adjuvanticity did not appear lessened in mice lacking fibrinogen, which is essential for alum to form nodular structures at the site of injection,^10^ again casting doubts on the idea that antigen retention is the primary mechanism. More defined biochemical analyses in recent years indicate that alum in other forms, such as hydrogels, binds to antigens via weak interactions and can be easily modified by biological fluids to lose adhesion. Some work went further to suggest that the intensity of the association between antigen and alum is inversely related to immunological outcomes.^11, 12, 13, 14^ These results challenged the ‘depot theory' and suggested other mechanisms at work. Certainly, these discussions on the depot effect started before the modern concept of antigen presentation. Current models are necessary to incorporate an immunological understanding of how alum mediates any facilitating effect in multiple steps from antigen uptake to final B-cell antibody production.^15^

### Role of the inflammasome

Although the depot theory has been discarded, the concept of adjuvant-mediated enhanced antigen presentation by antigen-presenting cells is now central to theories regarding adjuvant effects at large. Although signaling via toll-like receptors is regarded as the essential pathway for microbial pattern-based adjuvants,^16^ such as CPG (oligodeoxynucleotides), lipopolysaccharide and its derivatives, two independent studies showed that alum did not function through toll-like receptors.^17, 18^ Another class of intracellular pattern recognition receptors, the NOD-like receptors (NLRs), was also found to sense stimuli of microbial origin and endogenous signs of cellular damage.^19^ These receptors, upon activation, typically associate with an adapter molecule ASC (apoptosis-associated speck-like protein containing a caspase recruitment domain) and caspase-1 to form the oligomerized platform of an inflammasome that results in the auto-proteolysis of procaspase-1 to a generated active P10/20 dimer of activated caspase-1.^20^ The best-characterized activity of the inflammasome/activated caspase-1, in addition to mediating several forms of cell death, is the conversion of several inflammatory cytokines from their immature precursors to their active forms, including interleukin-1β (IL-1β), IL-18 and IL-33. Among them, the NLRP3 inflammasome is known to respond to particulate structures by producing IL-1β and IL-18.^21^ The exact signaling events are not well established. It was initially suggested that the signaling events derived from phagolysosomal destabilization; this proposal is now under debate because subsequent reports did not reproduce the same finding.^22, 23, 24^ Currently, Ca2^+^ influx, K^+^ efflux and mitochondria-originated reactive oxygen species are competing proposals.^24, 25, 26, 27, 28^

The role of the NLRP3 inflammasome in alum's adjuvanticity was reported. Using genetic mutant mice, Eisenbarth *et al.*^29^ indicated that antibody production induced by an alum/antigen mixture required NLRP3 (called Nalp3 at the time), ASC and caspase-1, in line with the established fact that macrophages and bone marrow-derived dendritic cells (DCs) from mice deficient in NLRP3, ASC or caspase-1 failed to produce IL-1β/IL-18 on stimulation with multiple types of aluminum adjuvants. Antibody production was therefore linked to IL-1β production or was thought to be critically dependent on an effector function downstream of NLRP3 activation because alum did not induce the production of IL-6 or tumor necrosis factor-α (TNFα) by primary macrophages *in vitro*. These manifestations often result from NFκB activation associated with microbial adjuvants.^30, 31^ In a somewhat unexpected finding immediately following Eisenbarth's report, Tschopp's group suggested that while phagocyte activation, caspase−1 conversion and IL-1β production were indeed observed in antigen-presenting cells following alum treatment, the effect paradoxically reduced antibody production of immunoglobulin G1 (IgG1) and IgG2c subclasses, with only IgE production relying on the NLRP3 platform.^32^

This disparity observed in earlier days is increasingly leading toward the consensus that NLRP3 is unlikely to be an indispensable component of alum's adjuvanticity, at least by the measurement of antibody production. Franchi *et al.* reported that deficiency in NLRP3 did not affect the production of antigen-specific IgG, IgA or IgM in an alum-based vaccination protocol.^33^ In addition, Marrack *et al* found caspase-1-deficient mice to have a sufficient antibody response. Importantly, in its absence, endogenous CD4 responses and their Th2 bias remained comparable to the wild type.^34^ Flach *et al.*^35^ used gene-deficient bone marrow DCs to show that while IL-1β production was diminished in the absence of NLRP3/ASC, the expression of TNFα and the co-stimulatory molecules CD80, CD86 and CD40 were not affected, and antibody production was even slightly increased. Collectively, evidence is increasingly dissociating the signaling cascade of NLRP3 and its effector functions from critical regulatory events leading to antibody production. The controversy may have resulted from different experimental setups and variability in protocols, preparation and other imperfectly controlled factors. In addition, antigen production reveals only one aspect of adaptive immune activation. The mere fact that alum is universally reported to trigger NLRP3 inflammasome activation suggests that there are immunological consequences yet to be revealed in the future.

### Roles of salts and crystals

In 2003, it was reported that uric acid is a principal endogenous danger signal released from injured cells during an attempt to identify a molecular base for the popular danger hypothesis.^36, 37^ The report noted that uric acid must crystalize to have any immune stimulatory effect. From the days of discovery of antigen cross-presentation, the immune regulatory functions of crystalline structures have been well documented;^38^ even though aluminum salts frequently form only amorphous clusters of small crystals, crystalline properties of alum were found to be central to its adjuvanticity. Lima *et al.*^39^ suggested that the primary result of crystal phagocytosis is the induction of cell death. This observation, coupled with the lack of involvement of the NLRP3 inflammasome, has led to two divergent theories regarding alum's mechanism of action. One theory suggests that alum adjuvanticity is a consequence of cell death, which, due to the brutal rupture of plasma membranes, is necrotic in nature and therefore proinflammatory.^40^ The other theory relies on the possibility that some cells may survive contact with alum and gain an increased ability for antigen presentation, which may lead to antibody production.

Intriguingly, new evidence obtained in more recent years points exactly in these two directions. Regarding the former, Marrack's group first reported that alum injection resulted in DNA release at the site, presumably from dead cells.^41^ Noting a similar observation of DNA release at the site of alum injection, Ishii's group revealed mechanistic insight into how the extracellular availability of DNA regulates antibody production from two different routes. First, DNA triggers IgG1 production. In parallel, DNA controls Th2 responses, promoting IgE isotype switching through signaling via the Tbk1/Irf3 axis.^42^ The study did not address the involvement of additional pathways regulated by DNA sensing; most prominent among them was AIM2 inflammasome-based caspase-1 activation^43, 44^ and STING/cGAS-mediated type I interferon release.^45, 46, 47^ Although the former's main effector function identified so far is caspase-1 activation, which may appear less attractive in light of controversies surrounding NLRP3's effector function, the latter is a direct activator of antigen-presenting cells. Interestingly, a visit to the latter yielded some unexpected intricacies.^48^ McKee *et al.*^48^ reported that in intramuscular immunization, although STING-deficient mice had reduced antigen-specific CD4^+^ T-cell activation and IgE production, IgG1 levels were not affected, in sharp contrast with the dramatic reduction of alum's adjuvant effect in the presence of DNase. Because STING is upstream of Tbk1/Irf3, the outcomes suggest that some other mechanisms triggered by released DNA are responsible for IgG1 production. In the same paper,^48^ the authors reported that removal of DNA did not alter the arrival of antigen-containing cells in draining lymph nodes but greatly reduced the steady contact between DCs and T cells, which likely resulted from reduced antigen presentation following DNA digestion. These effects indicate that the effects mediated by alum-triggered DNA release are still incompletely understood.

An additional proposal coincidentally links alum to the very production of uric acid. When aluminum salts and antigen were intraperitoneally injected into mice, the local concentration of uric acid was substantially increased.^49^ When mice were pre-treated with uricase (i.e., an enzyme to digest uric acid to allantoin) to degrade uric acid, CD4^+^ T-cell priming was inhibited. Here the adjuvant activity of aluminum salts does not require either Myd88 or IL-1β/IL-1 receptor engagement *in vivo*. This result is unexpected as uric acid crystals promote cross-presentation that enhances CD8^+^ T-cell responses in a seemingly opposing direction to CD4^+^ T-cell responses, which are required to boost B-cell activation.^37, 50^ A conceptual gap present in these discussions is how crystal-mediated cross-presentation, mainly a CD8^+^ T-cell phenomenon, turns on B-cell activation.^51^

In 2011, by means of atomic force microscopy, it was shown that independent of the inflammasome and membrane proteins, alum crystals bound to DC plasma membrane lipids with substantial force, subsequently activating an abortive phagocytic response that led to antigen uptake. Such activated DCs, without further association with alum, showed high affinity and stable binding to CD4^+^ T cells via the adhesion molecules intercellular adhesion molecule-1 and lymphocyte function-associated antigen-1. It was proposed that alum triggers DC responses by altering membrane lipid structures leading to the immune potentiating effects.^35^ This proposal attempts to address alum's effect from a biophysical point of view. How much this mechanism is of relevance to the total effect of alum's adjuvanticity requires further analyses.

## ALUM IN INFECTION CONTROL AND TUMOR SUPPRESSION

### Roles of alum in persistent infection

Thus far, studies on the functions of alum have only been conducted in cell lines and mice. Because more defined assays are available in these systems, theories regarding alum's mechanism of action in human vaccination are likely to be derived from these models. However, human trials are not without their own surprises. Recently, in a clinical trial employing HBV surface antigen (HBsAg)-human anti-HBs immunoglobulin immune complex (YIC) as a therapeutic vaccine for chronic hepatitis B virus (CHB) infection,^52, 53^ alum was used as an adjuvant and was inoculated alone as a negative control. The results for the first time revealed immunological effects of bare alum in humans. In the phase II B clinical trial, 74 CHB patients with a viral load >100 000 copies/mL and a serum ALT (alanine transaminase) of two to ten times the upper limit of normal were injected with 1 mL 0.1% alum emulsion intramuscularly once every four weeks, for a total of six injections. These patients were followed for 24 weeks after completion of immunization. Although the results showed significant differences between the YIC-immunized group and the alum immunized group, the HBeAg seroconversion (a criterion used to measure host response rates to immunization in clinical studies on viral hepatitis B) in the alum immunized group was 9%, higher than the spontaneous HBeAg seroconversion rate.^53^ When the number of injections increased to 12 and occurred over a 24-week period, surprisingly, among the 108 CHB patients injected with alum, the HBeAg seroconversion rate increased to 21%.^54^ To explore the possible mechanisms mediated by multiple injections of alum, mice were injected six times with alum alone or alum-adsorbed proteins (HBsAg-anti-HBs) at weekly intervals. After four injections, Gr1^+^/CD11b^+^ cells in the spleen were increased in both alum alone and alum/protein groups. After six injections, Gr1^+^/CD11b^+^ cells in the spleen remained consistently high in the alum alone-injected group, whereas Gr1^+^/CD11b^−^ cells decreased in the alum/protein group. Both groups showed increased levels of TNFα, but only the alum alone mice showed increased levels of IL-10. The histology of the liver tissues revealed a higher number of spotty necrotic foci in the alum alone group than in the controls.^55^ These outcomes reveal that alum alone could induce potent inflammatory responses and a sustained increase in cell-mediated immune responses. The underlying immunological regulations are yet to be studied. Nonetheless, these observations might promote using alum alone or in combination with other components even in the absence of overt antigens to modulate responses to persistent infections.

### Roles of alum in antitumor effects

Immunotherapies against existing cancers include active, passive or immunomodulatory strategies. Active immunotherapies are aimed to increase the ability of the patient's own immune system to mount a response to recognize tumor-associated antigens and eliminate malignant cells, whereas passive immunotherapy involves administration of exogenously produced components, such as lymphocytes or antibodies, to mediate an immune response. Immunomodulatory agents are not targeted at specific antigens but are intended to enhance general responsiveness and amplify anticancer immunity.^56^ Because alum has been shown to induce both inflammatory responses and trigger cell-mediated immune responses, Wang *et al.*^57^ explored whether alum has any effects on tumors. After multiple trials, a specific protocol to induce antitumor effects by alum-only therapy was identified. Balb/c mice with established H22 hepatocarcinoma were immunized with multiple intraperitoneal injections of alum after five, seven or ten days. Interestingly, only when alum treatment was initiated five days (5 DPI) after the establishment of the tumor was significant growth reduction detected; the differences became more distinct over time.^57^ Cytokine profiles in peritoneal lavage and tumor homogenate were followed; they showed that IL-1β and TNFα were increased in the alum-treated groups, suggesting that the presence of particulate or crystalline structures can activate innate immune responses, particularly as a consequence of interaction with phagocytes associated with an enhanced inflammatory response. To study whether alum-mediated tumor suppression also requires adaptive immunity, nude mice were used in a similar study, and the benefit of alum was completely lost. The authors further studied the roles played by infiltrating neutrophils, CD4^+^ and CD8^+^ T cells, and chemokines. It was found that CD8^+^ T cells were essential to alum-associated tumor suppression. In this pilot study, it was not clear why the alum-only treatment was effective only at 5 DPI in this tumor model, and it was postulated that the lack of effect at the other immunization times suggests that the alum 5 DPI treatment captured a window in tumor establishment when it was penetrable to immune surveillance, which disappeared at later time points. This result suggests that each individual immunotherapy may have an effective time point to be employed. Because immunotherapy works more effectively in personalized medicine, studies regarding each type of immunotherapy should be fully explored in this regard.

## SUMMARY AND PERSPECTIVES

Alum has long been used as an adjuvant, and its efficacy and safety are proven. It has been shown to enhance antibody responses as well as to stimulate innate immunity; for example, it induces the conversion of macrophages to antigen-presenting dendritic cells.^58^ A number of proinflammatory cytokines and chemokines are induced at the site of injection, and different types of cells including neutrophils, natural killer cells, natural killer T cells, eosinophils and DCs are recruited locally. Alum was found to induce endogenous CD4^+^ T cells and antibody production as well as to induce priming of CD8^+^ T cells. These effects are shown to be independent of the inflammasome. Furthermore, the direct interaction between alum and the cell surface lipids makes the functions of alum in hosts even more complex and interesting.

Although new adjuvants such as MF59 (ref. 59) and liquid crystals^60^ are evolving, alum, as the oldest adjuvant, has shown certain new effects yet to be explored. Using alum as a nonspecific stimulus to modulate host immune responses in persistent infections is an interesting frontier for future study. The novel finding of functions of alum in cancer therapy also needs further analysis. Currently, while facing multiple emerging infectious agents and diseases and the risk of bio-attack by terrorists, the world should be prepared to develop ‘first aid immunological regulators' or ‘nonconventional first aid vaccines' to address these unpredictable challenges. The idea is that when the emerging infectious agent has not yet been identified but the population urgently needs protection, a nonspecific immune stimulating substance, mimicking the ‘first aid' measures taken in clinical medicine, should be prepared and ready for use. Alum, with its low cost, stability, safety and stimulatory effects on the immune response, might be optimized and developed as one of the candidates for a future ‘nonconventional first aid vaccine'.
